# PCD Reflections on Progress and Successes Achieved in 2022

**DOI:** 10.5888/pcd19.220385

**Published:** 2022-12-08

**Authors:** Leonard Jack

**Affiliations:** 1 Office of Medicine and Science, National Center for Chronic Disease Prevention and Health Promotion, Centers for Disease Control and Prevention, Atlanta, Georgia

*Preventing Chronic Disease* (PCD) started 2022 by celebrating the largest Impact Factor increase in the journal’s history. The journal’s Impact Factor increased from 2.803 in 2020 to 4.354 in 2021. The journal also retained its ranking at 4th place out of 30 US journals and 76th out of 585 journals worldwide in the Public Health, Environmental and Occupational Health open access journal category. PCD achieved a new ranking in the top 10% of 27,339 journals evaluated by Scimago in 2021. The journal credits these impressive metrics to an outstanding group of volunteers serving as Associate Editors, Editorial Board members, and Statistics Review Committee members. The journal’s successes are also the result of a staff committed to constantly evolving, rethinking production processes, and improving the experiences of authors who seek to submit papers to the journal for consideration. In addition, the journal has also continued to receive unwavering support from CDC leadership, particularly senior leadership in the National Center for Chronic Disease Prevention and Health Promotion.

## 2022 PCD Collections

PCD has continued to identify timely topics, this year publishing 4 collections focusing on content of greatest interest to our readership. Three collections covered specific topics, and one, the Student Paper Contest, covered many topics by student authors and researchers.

In April 2022, PCD released the collection *Cancer Screening Prevalence and Associated Factors Among US Adults* (1). This collection of 11 articles presented research on cancer screening trends, determinants of cancer screening, and public health practices that increase cancer screening uptake in specific populations.

In June 2022, PCD released its collection *Geospatial Perspectives on the Intersection of Chronic Disease and COVID-19* (2). The 11 articles in this collection bring together scientists and practitioners from public health and social sciences to demonstrate how geospatial perspectives can contribute to understanding and addressing the complex ways in which the COVID-19 pandemic has affected the health of people with chronic disease.

PCD is the only peer-reviewed journal that annually offers student authors the opportunity to be recognized as winners in multiple categories for their quality research and to become first authors on a peer-reviewed paper. PCD’s annual Student Paper Contest welcomes papers generated by high school, undergraduate, graduate, doctoral, and postdoctoral students. Submissions to the journal in response to its Call for Papers are peer reviewed for publication, and winners may be selected from the education level categories. PCD released its most recent Student Paper Contest collection in November 2022 (3). Eight papers appear in this collection, focusing on topics such as COVID-19 and various aspects of health, food insecurities among caregivers in Southern states, the relationship between physical activity and depression among high school students, perceptions of neighborhood development on active living among community residents, use of a cancer index as a predictor of common cancers, and spatial analysis of breast cancer mortality rates in rural states.

PCD released its final collection in 2022, *Global Responses to Prevent, Manage, and Control Cardiovascular Disease* (4), in December. This collection featured peer-reviewed articles generated from authors around the world, offering insight into successes and challenges of public health strategies to improve cardiovascular health through prevention, detection, and treatment. The collection has 20 articles that examine factors contributing to the decrease or increase in heart disease risk, drivers of racial and ethnic minority population disparities in cardiovascular health, health equity approaches to reducing coronary heart disease and stroke among diverse populations, monitoring and tracking systems, and lessons learned from heart disease and stroke programs.

Also in 2022, PCD received and peer-reviewed papers in response to the journal’s call for papers *Health Equity in Action: Research, Practice, and Policy*. Advancing health equity and eliminating health disparities have been and continue to be critical areas of interest to PCD. *Healthy People 2030* defines health equity as the attainment of the highest level of health for people. “Achieving health equity requires valuing everyone equally with focused and ongoing societal efforts to address avoidable inequalities, historical and contemporary injustices, and social determinants of health — and to eliminate disparities in health and health care” (5). This PCD collection, scheduled for publication in March 2023, will feature articles focusing on integrating health equity in all policies; identifying potential health interventions to improve health equity in diverse populations and settings; integrating health equity in program design, implementation, and evaluation; and engaging community stakeholders to advance health equity.

## 2023 PCD Call for Papers

PCD is also looking ahead to the topics that are driving future research. In response, the journal has Calls for Papers in 5 key areas of research.

### Sleep Deprivation, Sleep Disorders, and Chronic Disease

Sleep is an essential daily behavior that supports physical, emotional, and psychological well-being. Nearly every system of the body depends on satisfactory sleep quality and quantity for routine healing, repair, and restoration. PCD is interested in publishing peer-reviewed papers that bring increased attention to the relationship between sleep and chronic disease and offer insight into successes and challenges of public health strategies to improve the quality of sleep. This Call for Papers closed on December 15; however, PCD continues to welcome papers in this less-explored and less-published area of public health.

### Advancing Chronic Disease Data Modernization Enhancements to Meet Current and Future Public Health Challenges

The nation’s health data systems are often antiquated, resulting in myriad negative effects on chronic disease prevention and health promotion efforts. In addition, antiquated data management systems often become difficult to support, maintain, scale, or integrate into new platforms. PCD is interested in publishing papers describing and highlighting data management infrastructure transformations that support response-ready systems capable of meeting current and future health challenges. Please visit https://www.cdc.gov/pcd/announcements.htm#capAdvMod to learn more about the data modernization Call for Papers.

### 2023 PCD Annual Student Paper Contest

PCD is continuing its tradition as the only peer-reviewed journal that annually recognizes students who are the first author on a peer-reviewed paper by selecting winners in multiple categories for their quality research. For 2023, PCD welcomes submissions for its annual Student Paper Contest from students at the high school, undergraduate, and graduate levels; recent postgraduates; and medical residents. PCD is interested in publishing papers relevant to the prevention, screening, surveillance, and population-based intervention of chronic diseases, including but not limited to arthritis, asthma, cancer, depression, diabetes, obesity, cardiovascular disease, and COVID-19 and chronic conditions. Please visit https://www.cdc.gov/pcd/announcements.htm#capStu2023 to learn more about the Student Paper Contest Call for Papers.

### Implementing and Sustaining Policy, Systems, and Environmental (PSE) Approaches to Support Healthy Behaviors in Diverse Settings

Over the past decade, there has been increased understanding that health behaviors are not only shaped by individual choices but also influenced heavily by the environments in which they occur. The use of policy, systems, and environmental (PSE) approaches has emerged as a possible avenue to reach populations by implementing a combination of strategies that create supportive conditions for individuals to adopt and sustain healthier behaviors to prevent or manage chronic disease. PCD is interested in receiving papers that focus on implementation and scaling up of novel PSE approaches and their impact on chronic diseases and risk factors. Please visit https://www.cdc.gov/pcd/announcements.htm#capImpSus to learn more about the PSE approaches Call for Papers.

### Tools and Techniques to Effectively and Suitably Conduct Program Evaluation

Evaluation is a key public health function used for monitoring and measuring the quality, pace, direction, and impact of interventions. PCD is interested in publishing peer-reviewed papers featuring the journal’s article type Tools for Public Health Practice (6). This article type provides instructional content to support professional development and focuses on the “how-to” practical application of public health methods in performance monitoring and program evaluation. Please visit https://www.cdc.gov/pcd/announcements.htm#capTools to learn more about the Tools for Public Health Practice Call for Papers.

## Commitment to Diversity, Equity, and Inclusion

Keeping PCD in the best position to publish relevant peer-reviewed articles requires that we continue our commitment to advancing diversity, equity, and inclusion (DEI) as well as best practices at all levels of operation. This year, PCD created a dedicated webpage that provides details on the journal’s commitment to diversity, equity, and inclusion. This page provides DEI-related resources such as diversifying editorial boards, collecting optional demographic data, incorporating inclusive language in scholarly communication, and sharing DEI statements in published papers that indicate how the authors’ work, their research group, or both are contributing to making public health science and practice more diverse, equitable, and inclusive. Please visit https://www.cdc.gov/pcd/about_the_journal/pcd_diversity.htm to learn more.

## Conclusion

PCD closes 2022 with much to celebrate: impressive improvements in its Impact Factor, higher ranking among peer-reviewed journals worldwide, new collections, and an outstanding team of experts and staff dedicated to bringing the very best in research and practice to the public health community. The journal’s 4 collections this year have been a great success, drawing media attention from around the world. This success would not have been possible without the engagement and participation of our expert volunteers, who have provided hundreds of hours reviewing and providing feedback on manuscripts submitted to the journal for consideration. The journal’s staff have continued to bring innovation to the journal’s day-to-day operations, often serving as a model for other journals in this area. Despite the continuing challenges of the pandemic, the number of submissions to the journal remained steady in 2022, and we anticipate this steady submission of papers will continue in 2023. On behalf of the journal’s entire staff, we thank our readers and volunteers for their tremendous support and look forward to an exciting and successful 2023.
